# Combined iPLEX and TaqMan assays to screen for 45 common mutations in Lynch syndrome and FAP patients

**DOI:** 10.1186/1897-4287-9-S2-A9

**Published:** 2011-06-01

**Authors:** D Dymerska, P Serrano Fernández, J Suchy, A Pławski, R Słomski, K Kąklewski, RJ Scott, J Gronwald, J Kładny, T Byrski, T Huzarski, J Lubiński, G Kurzawski

**Affiliations:** 1 International Hereditary Cancer Center, Department of Genetics and Pathology, Pomeranian Medical University, Szczecin, Poland; 2 Discipline of Medical Genetics, Faculty of Health, University of Newcastle and the Hunter Medical Research Institute, Newcastle, NSW, Australia; 3 Division of Genetics, Hunter Area Pathology Service, John Hunter Hospital, Newcastle, NSW, Australia; 4 Institute of Human Genetics, Polish Academy of Sciences, Poznan, Poland

## Background

It is now well recognized that somewhere between 10 and 20% of all colon cancers are a result of a highly penetrant genetic predisposition. The most commonly identified types of hereditary colorectal cancer are hereditary non-polyposis colorectal cancer (HNPCC or Lynch syndrome) which accounts for an estimated 2-5% of all colorectal cancer patients and familial adenomatous polyposis (FAP) which contributes to about 1% of them. The occurrence of Lynch syndrome (LS) is associated with constitutional mutations in DNA mismatch repair (MMR) genes. FAP is a result of inactivating germline mutations in the adenomatous polyposis coli (*APC*) gene. An iPLEX test combined with TaqMan genotyping assays was developed to identify common recurrent mutations of those genes in the Polish population.

## Materials and methods

349 DNA samples from 95 positive controls, identified by sequencing and 254 individuals not tested previously by any method, were analysed. The iPLEX test included two plexes, comprising 7 mutations of the *APC* gene and 29 mutations of three MMR genes: *MLH1*, *MSH2* and *MSH6*. TaqMan assays were designed for 9 mutations not covered by iPLEX assays, one mutation in *APC* gene and 8 mutations in three MMR genes. Results were verified independently by sequencing to determine the sensitivity and specificity for iPLEX/TaqMan test in a blind study.

## Results

Our combined method allowed to detect all recurrent mutations occurred in group of patients, which was subjected to full analysis by DNA sequencing. With the exception of one false positive in iPLEX test in the group of positive controls, that could be assigned to contamination from neighbouring wells rather than a detection error, given sufficient DNA concentration and quality, the designed iPLEX/TaqMan test had an accuracy of 100% for the designed assays.

## Conclusions

Results suggest that the combined iPLEX/TaqMan test is an outstanding tool for identification of recurrent mutations among hereditary colorectal cancer patients. An extension of the test to all known MMR and *APC* mutations, and not just the ones currently considered as recurrent, should be also taken into consideration.

